# Synovial sarcoma of the head and neck: A review of reported cases on the clinical characteristics and treatment methods

**DOI:** 10.3389/fcell.2022.1077756

**Published:** 2023-01-06

**Authors:** Hongzhi Quan, Senjeet Sreekissoon, Yan Wang

**Affiliations:** ^1^ Department of Oral Maxillofacial Surgery, Xiangya Stomatological Hospital and School of Stomatology, Central South University, Changsha, Hunan, China; ^2^ School and Hospital of Stomatology, Wenzhou Medical University, Wenzhou, Zhejiang, China

**Keywords:** synovial sarcoma of the head and neck, diagnosis, treatments, clinical manifestations, management

## Abstract

Synovial sarcoma (SS) is a high-grade soft-tissue sarcoma that occurs predominantly in older children and young adults in their thirties. It is usually very challenging to diagnose and treat synovial sarcoma in the head and neck region. The purpose of this review is to investigate the clinical manifestations and different treatment methods in the management of primary synovial sarcoma of the head and neck. HNSS has an aggressive nature and poor prognosis. Surgical resection, radiotherapy, and chemotherapy are the primary treatment methods. Typically, surgical resection with negative margins remains the foundation of therapy, which is not very easily achieved in the head and neck due to its complex anatomical structure and the presence of many blood vessels and nerves. However, synovial sarcoma has a high recurrence rate, so aggressive management and close follow-up are warranted for the optimal outcome.

## Introduction

Jernstrom (1954) first described synovial sarcoma (SS) in 1954. SS is a soft tissue malignancy harboring t (X; 18), resulting in the fusion of two genes SS8 (at 18q11) and SSX (1, 2, or 4 at Xp11), forming the gene fusion product SS18–SSX (Stacchiotti and Van Tine, 2018). It primarily affects young adults in their thirties and most frequently occurs in the para-auricular regions of the extremities (70%), followed by the trunk (15%), and least common in the head and neck region (5%–7%) (Sultan et al., 2009; Stacchiotti and Van Tine, 2018). SS of the head and neck (HNSS) is a rare, aggressive malignant tumor with an unpredictable prognosis and is prone to recurrence after treatment (Kumar et al., 2020). The most common site of HNSS is the hypopharynx (Pai et al., 1993). However, HNSS also arises from other sites, including the oropharynx (Herrero Laso and Varela Duran, 1998), nasopharynx (Nakahira et al., 2013), trachea (Reilly and Johnston, 2010), TMJ (Xia et al., 2020), mandible, tongue, paranasal sinuses, floor of the mouth, buccal mucosa (de Araujo and Monteiro, 1989; Mahesh et al., 2013), maxillary sinus (Hannoun et al., 2021), ethmoid sinus (Jain et al., 2018), parotid gland (Grayson et al., 1998), soft and hard palate (Massarelli et al., 1978; Ferlito et al., 1981; Doubi et al., 2019), gingiva (Rao et al., 2014), retromolar area (Meer et al., 2003), and suboccipital region (Karydakis et al., 2018).

**FIGURE 1:**
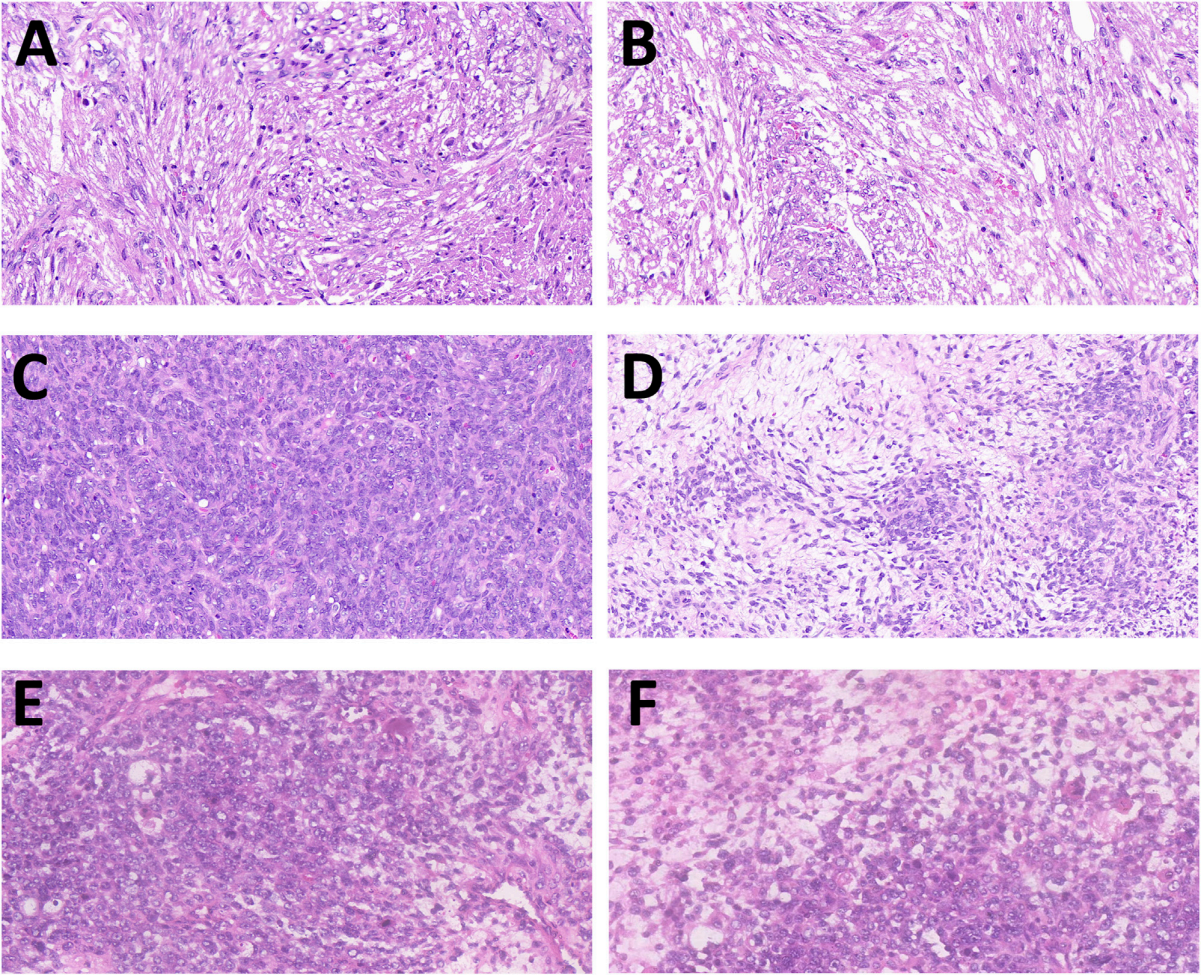
Hematoxylin and eosin staining of the three histologic variants of synovial sarcoma (SS). **(A,B)** Slides of monophasic SS, made of spindle cells with moderate cytologic atypia and differentiated areas of variable cellularity. **(C,D)** Slides of biphasic SS composed by glandular-like structures with spindle cells without nuclear atypia. **(E,F)** Slides of poorly differentiated SS in its Ewing-like variant, with the presence of rosette-like structures (magnification: ×20).

According to the International Classification of Diseases for Oncology (ICD-O), SS is divided into three distinct histologic subtypes: monophasic SS, biphasic SS, and poorly differentiated SS (Fiore et al., 2021). Figure 1 shows hematoxylin and eosin staining of the three histologic variants of SS. Other variants include myxoid SS and ossifying/calcifying SS (Shukla et al., 2003; Alabdulaaly et al., 2021). Monophasic SS contains uniform spindle cells, biphasic SS consists of epithelial cells arranged into glandular structures with spindle cells arranged into fascicles, and spindles and round blue cells characterize poorly differentiated SS (Jayasooriya et al., 2016). The monophasic subtype and the biphasic subtype are the two most common subtypes, while the epithelioid cell subtype is rare. Histologically, SS comprised varying proportions of spindle and epithelial cell components. Due to the variable cellular and architectural morphology and resemblance to other neoplastic processes common to the region, the histopathological diagnosis of SS is very challenging (Crowson et al., 2015). Immunohistochemistry plays a crucial role in histological diagnosis. SS is positive for epithelial markers, including cytokeratin, epithelial membrane antigen (EMA), and vimentin. SS is usually unfavorable for CD34 and FLI-1 (Madabhavi et al., 2018).

The causes and risk factors of SS remain unclear. Researchers have found that in radiotherapy of other cancers, specific inherited gene defects can increase the possibility of getting SS (Ulusan et al., 2005). Furthermore, other studies suggested that SS can be derived from undifferentiated cells, neural crest stem cells, pluripotent mesenchymal cells, and other parts of the body (Carrillo et al., 1992; Machen et al., 1999; Sturgis and Potter, 2003; De Logu et al., 2020). This review about HNSS was written to understand better different treatment methods of SS in the head and neck region in many cases reported before due to the rarity of the disease and lack of data. It is imperative to know how to manage and treat HNSS to improve the health status of patients suffering from SS in the head and neck region.

### Synovial sarcoma in laryngeal and pharyngeal regions

The first case of HNSS, which occurred in the pharynx, was described in 1954 by Jernstrom (1954). It is also the most common site HNSS (Pai et al., 1993). The most common signs and symptoms of SS in the laryngeal and pharyngeal region among patients are hoarseness of voice because of proliferative growth involving the vocal cord and aryepiglottic fold with impaired mobility (Kumar et al., 2020), hemoptysis (Papaspyrou et al., 2003; Madabhavi et al., 2018), dyspnea, dysphagia, mass in the neck region, breathing difficulties, and pain. Sometimes the patients are misdiagnosed, such as fibroadenoma or benign tumor, because of the rarity of SS in the head and neck region and lack of clinical and imaging information (Danninger et al., 1994; Wigand et al., 2018; Kaoutar et al., 2021). CT, MRI, and PET-CT can be used in the examination of the patients, and smaller-size SS often shows similar imaging features similar to benign tumors displayed by CT or MRI (Hirsch et al., 1997; Rangheard et al., 2001; Hongzhi Quan et al., 2017).

As shown in Table 1, surgery is the principal treatment modality for synovial sarcoma of the larynx and pharynx, including partial or complete laryngectomy, partial laryngectomy, endoscopic resection, neck dissection, laser endoscopic resection, and CO_2_ laser excision (Pruszczynski et al., 1989; Capelli et al., 2007). Neck dissection is unnecessary if the tumor does not involve the lymph node (Meer et al., 2003). In some cases, surgery is performed following radiotherapy and chemotherapy, while others undergo surgery followed by radiotherapy without chemotherapy, depending on the patient’s physical status and the physician’s advice. From Table 1, we can see that numerous patients underwent laser endoscopic resection, laryngectomy neck dissection, extended total laryngectomy, and CO_2_ laser excision, followed by radiotherapy and chemotherapy. Some chemotherapy agents are ifosfamide, adriamycin, cisplatin, actinomycin D, vincristine, dacarbazine, doxetaxel, rh-endostatin, dosorumin, and others (Shein et al., 2021). Table 1shows that the patient underwent postoperative radiotherapy delivering 60 Gy in 30 fractions over 6 weeks, with bilateral prophylactic nodal irradiation to 50 Gy (Holtz and Magielski, 1985). Then, the patient was administered four cycles of doxorubicin and ifosfamide chemotherapy. Twelve months after the initial diagnosis, there was no evidence of recurrence on endoscopy (Madabhavi et al., 2018). In most cases described as follows after treatment of SS, the recurrence rate is quite low, and the patients were cured.

**TABLE 1:** Reported cases of laryngeal and pharyngeal synovial sarcoma.

Reference	Age/sex	Location	Size (cm)	Signs and symptoms	Type	Treatment	Follow-up
Papaspyrou et al. (2003)	16/M	Right aryepiglottic fold	4 × 3 × 3	Dysphagia for 3 mo accompanied with hemoptysis, lost 6 kg in 2 mo, and change of voice in the past 15–20 days	Biphasic	Laser endoscopic recs + R	NED after 2 years
Madabhavi et al. (2018)	31/M	Left arytenoid and left pyriform fossa	8 × 4 × 3	Hoarseness of voice since 5 weeks	Unk	S, post-operative R and C	NED after 12 mo
DPC and MacGregor (2012)	11/M	Right aryepiglottic fold	Unk	Unk	Unk	Pre-operative C + R and total laryngectomy	NED after 6 years
Hongzhi Quan et al. (2017)	17/M	Right neck	8 × 6.5	Unk	Biphasic	Surgical resection	Disease-free more than 18 mo
Pruszczynski et al. (1989)	28/F	Left aryepiglottic fold and false cord	2 × 3	Progressive inspiratory stridor of 6 mo and a change of voice of 2 mo and progressive dysphagia	Monophasic	Endoscopic recs + R	NED after 3 years
Ferlito et al. (1981)	28/M	Right aryepiglottic fold and epiglottis	2.5 × 3	Progressive dysphagia and intermittent right otalgia for 2 mo	Biphasic	R pre and post-operative, supraglottic, laryngectomy, and right neck dissection	NED after 16 years
Danninger et al. (1994)	53/M	Right aryepiglottic fold and pyriform sinus	Unk	Unk	Unk	Total laryngectomy and neck dissection + R	NED after 16 mo
de Almeida-Lawall et al. (2009)	68/F	Cricoid cartilage	Unk	Slowly enlarging neck mass for 5 mo and difficulty in breathing over the last 2 weeks	Biphasic	Extended total laryngectomy and neck dissection	—
Bilgic et al. (2003)	24/M	Left aryepiglottic fold, epiglottis, and left arytenoid	3 × 2.5 × 0.7	Changing of his voice and dysphagia for several mo	Biphasic	Hemilaryngectomy, total laryngectomy, neck dissection, and R + C	Recurrence after 1 year Lung metastasis after 20 mo NED after 3.5 years
Wigand et al. (2018)	26/M	Epiglottis	2.42 × 2 × 1.5	Suffered from insidious and slowly progressive odynophagia for 5–6 mo	Biphasic	Laser endoscopic resection neoadjuvant C, partial laryngectomy, and postoperative R + C	Recurrence after 6 weeks NED after 20 mo
Capelli et al. (2007)	57/M	Left laryngeal ventricle	Unk	Dysphonia for few mo	Monophasic	CO_2_ laser excision	NED after 14 mo
Fonseca et al. (2014)	23/F	Pharyngeal tumor in the left posterolateral wall near the base of the tongue	5.3 × 3.9 × 2.2	Dysphagia and difficulty of breathing for 8 mo, resulting in progressive worsening and emergence of snoring, muffled voice, and local pain	Unk	Endoscopic pharyngectomy was used to excise the tumor. C + R	1 year postoperatively, the patient continues with locoregional control without recurrence
Kaoutar et al. (2021)	23/F	Posterolateral pharyngeal wall	6	Cervical algia and left otalgia since 1 year	Unk	C + R	Unk
Holtz and Magielski (1985)	16/M	Mid-line above the hyoid bone	6 × 4 × 4	Sore throat, fever, and a mid-line suprahyoid mass. Midline enlargement above the hyoid bone persisted	Biphasic	S + R (6,700 rad) given over 8 weeks. 12 mo C course of monthly vincristine, dactinomycin, cyclophosphamide, roentgenographic, and hématologic	6 mo later, metastasis was detected at the right pericardiophrenic angle involving the lower lobe of the right lung, the diaphragm, and the pericardium. Patient died 37 mo

Unk, unknown; Recs, resection; S, surgery; R, radiotherapy; C, chemotherapy; mo, month; NED, no evidence of disease.

### Synovial sarcoma of the oral cavity (TMJ, jaw, and tongue)

SS of the oral cavity region is not very common compared to that of the pharyngeal and laryngeal regions. Based on literature, the commonly affected sites of the oral cavity include the jaw, TMJ, and tongue. Table 2 shows SS of the oral cavity (TMJ, jaw, and tongue) (Maxymiw and Wood, 1990; Tilakaratne, 2006; Wang et al., 2008). The clinical symptoms of SS in the TMJ include pre-auricular mass, mouth opening limitation, discomfort in the TMJ, trismus, pain, and swelling in the face (Allias-Montmayeur et al., 1997). SS in the TMJ region may often be misdiagnosed as temporomandibular disorder (TMD) or other benign neoplasms due to the typical slow growth and signs and symptoms (Mostafapour and Futran, 2000). Differential diagnosis, including primary parotid tumor, should also be considered. Before surgery, CT scan and panoramic radiography can also be very helpful in revealing the size and location of the tumor (Bukawa et al., 2007). The ratio of occurrence of SS in males to females in the TMJ region is 8:5. In the current review, surgical excision in the TMJ region was performed in all cases. Local excision was performed through a standard parotid incision with temporal extension (Madabhavi et al., 2018). The metastasis rate of the lymph node is 10%–20%, and prophylactic cervical lymph node dissection is not necessary (Kartha and Bumpous, 2002). In the TMJ region, radical excision with negative margins is not always successful due to the complex anatomical structure. In such cases, surgical excision with adjuvant radiation has shown positive effects and an improved overall survival rate in patients (Stadelmann et al., 1995; Harb et al., 2007). Although the effectiveness of chemotherapy is still controversial, it has been tried in some cases (White et al., 1992; Meer et al., 2003; Luo et al., 2007; Al-Daraji et al., 2009; Nomura and Kishimoto, 2014). Patients with a tumor 5.0 cm in diameter had a higher risk of local tumor recurrence, distant metastasis, and mortality in HNSS (Gopalakrishnan et al., 2017). Other prognostic factors include age, TMN stage, surgical margin, and therapy modality.

**TABLE 2:** Reported cases of the oral cavity (TMJ, jaw, and tongue).

Reference	Age/sex	Location	Size (cm)	Signs and symptoms	Type	Treatment	Follow-up
Xia et al. (2020)	32/F	TMJ region	2.9 × 2.5 × 3.4	Pre-auricular mass, mouth opening limitation, and tenderness	Biphasic	S + R + C	6 mo NED
Bukawa et al. (2007)	67/M	TMJ region	5 × 4.2 × 3	Chronic discomfort, trismus, and TMJ pain	Monophasic epithelial	S	24 mo died of other disease
Mostafapour and Futran (2000)	23/F	TMJ region	3 × 4 × 3	TMJ syndrome, mild trismus, a right infra- temporal non-tender mass, and decreased sensation along the third division of the right trigeminal nerve	Monophasic	S + R	54 mo alive NED
Mostafapour and Futran (2000)	35/F	TMJ region	Unk	TMJ pain and left facial swelling	Monophasic	S + R	18 mo DOD
Wang et al. (2008)	32/M	TMJ condyle process	5 × 4	Pre-auricular mass and mouth opening limitation	Biphasic	S	Unk
Luo et al. (2007)	21/F	TMJ region	4 × 3	Tender mass over the right pre-auricular region, discomfort over TMJ, tenderness, and mouth opening limitation	Biphasic	S + R + C	60 mo and NED
White et al. (1992)	57/M	TMJ region	Unk	TMJ pain, right posterior open bite, slight swelling, and mandibular deviation	Biphasic	S + R + C	15 mo DOD
Al-Daraji et al. (2009)	61/M	TMJ region	1	Unk	Monophasic	S	18 mo
							DOD
Al-Daraji et al. (2009)	35/F	Coronoid process TMJ	4	Unk	Monophasic	S + R + C	201.6 mo
							Alive and NED
Al-Daraji et al. (2009)	22/M	TMJ region	5 × 4	Unk	Monophasic + poorly differentiated	S	9.6 mo
							DOD
Allias-Montmayeur et al. (1997)	39/M	TMJ region	2 × 2	Pre-auricular swelling, mouth opening limitation, and mandibular deviation	Biphasic	S	5 mo alive NED
Stadelmann et al. (1995)	49/M	TMJ region	3 × 1.5 × 1.5	Painless pre-auricular mass and chronic and severe TMJ pain	Biphasic	S + R	9 mo alive NED
Nomura and Kishimoto (2014)	17/M	TMJ region	5.5	Pain in the TMJ	Unk	S + R + C	62 mo DOD
Teixeira et al. (2021)	22/M	Lesion, extending from the left condyle to the mandibular body	Unk	Pain in the region of the left mandibular body and ramus	Monophasic	S	Last 2 years
							NED
Granowetter et al. (2006)	11/M	Mandible	Unk	Left lower jaw pain and increasing difficulty in eating due to the inability to fully open his mouth	Monophasic	C (vincristine, actinomycin-D and cyclophosphamide, and ifosfamide, vincristine, and doxorubicin) +R + S	3 years alive
Tilakaratne (2006)	76/M	Mandible	Unk	Progressive swelling in the left side of his face of 1 mo duration	Monophasic	Hemimandibulectomy with levels I–III cervical lymph node dissection	Patient died after 2 mo (extensive metastasis to the lungs)
Tao et al. (2011)	20/F	Mandible	Unk	Recurring swelling in the left posterior region	Monophasic	S (mandibulectomy and lymph node dissection) +R (4,000 cGy), for 1 mo	1 year alive
				Teeth, without pain, but she felt alveodental suppuration			
Tilakaratne (2006)	29/F	Mandible	Unk	Bony hard swelling over the right ramous of the mandible for 2 mo and gradually increase in size	Monophasic	S	2 years alive
Maxymiw and Wood (1990)	32/F	Maxilla	Unk	Right facial mass	Biphasic	C + R	Dead
Alabdulaaly et al. (2021)	14/F	Tongue, floor of the mouth, and lingual gingiva of the anterior mandibular teeth	7.4 × 4.7 × 5.6	Painful tongue mass growing over past 3 years and difficulty eating solid food. Loss of ability to speak and unable to close her lips	Monophasic and calcifying	R (75 Gy) + C (doxorubicin, ifosfamide, and pazopanib)	13 mo. Patient alive with disease. NEM
Villaroel-Salinas et al. (2012)	31/F	Based on the lateral pharyngeal wall and the base of the tongue	3.4 × 2.3	Progressively growing tumor in the base of the tongue	Monophasic	S	Unk
Sharma and Mehan (2019)	22/M	Base of the tongue	Unk	Unk	Monophasic	Unk	Unk
Novotny and Fort (1971)	21/M	Base of the tongue	3.5	“Fullness in the throat” for 1 mo + difficulty in speech + slight hoarseness + difficulty in swallowing, with sticking of food and frequent choking	Monophasic	R	NED (2 years)
Bridge et al. (1988)	31/M	Base of the tongue	2.5	Unk	Biphasic	S	Unk
Carrillo et al. (1992)	13/M	Lateral tongue (left)	1.0	Swelling in the left	Biphasic	S	Patient is alive and well 1 year following excision
				Lateral aspect of the tongue			
Su et al. (2018)	49/M	Posterior one-third (left)	Unk	Painful bleeding mass located in the left side of the tongue of 4 mo duration	Biphasic	S	Patient remains Symptom-free 2 years postoperatively
de Almeida-Lawall et al. (2009)	26/M	Posteriolateral aspect of the tongue (right)	1	Unk	Biphasic	S	18 mo, patient well, with no signs of tumor activity
Su et al. (2018)	35/M	Posterior one-third (left)	Unk	Mildly painful and non-mobile	Unk	S + R	Every 2 mo for 2 years and then every 6 mo for 2 years
				Lump on the left side of his tongue 1 year			
Engelhardt and Leafstedt (1983)	25/M	Mass in the right tonsil, attached to the anterior tonsillar pillar and at the tongue base	5 × 3	Discomfort in the posterior oral cavity	Unk	S (tracheostomy), and reconstruction *via* transmandibular symphysis osteotomy	NED (38 mo)
Bridge et al. (1988)	31/M	Right base of the tongue with encroachment on the epiglottis	2.5	2-month sore throat, intermittent otalgia, dyspnea, and occasional hemoptysis	Biphasic	Laryngectomy with a right radical neck dissection	Unk
				Sought medical attention after experiencing a sudden choking sensation and coughing up an ovoid mass of necrotic tissue			
				Approximately 2.5 cm in diameter			
Sharma and Mehan (2019)	74/F	Right lateral border of the tongue	5 × 1 × 3	Dysphagia (3 mo)	Monophasic	S + R	Disease-free
							During 2 years of follow up

Unk, unknown; Recs, resection; S, surgery; R, radiotherapy; C, chemotherapy; mo, month; NED, no evidence of disease; DOD, died of disease.

SS in the maxilla and mandible is infrequent compared to that in the TMJ. In this region, SS is usually diagnosed in young female patients, with the mandible and maxilla equally affected (Liu et al., 2015) (three males and three females). Table 2 shows the clinical symptoms of SS of the jaw, including painful swelling, limitation in mouth opening, eating difficulty, and swelling of the face. The diagnosis of intraoral SS and radiographic examination are essential steps used to localize and identify the lesion such as in Table 2 intraoral examination, an expansion of the cortical bone was noticed in the vestibular and lingual regions of the left mandible, and radiographic examination showed the presence of an expansive and multiocular radiolucent lesion extending from the left condyle to the mandible body (Granowetter et al., 2006; Teixeira et al., 2021). The clinical diagnostic hypotheses were ameloblastoma or malignant neoplasm. A combination of clinical, histologic, and immunohistochemical approaches can be used to support the diagnosis of SS in the jaw.

In the jaw region, biopsy is usually performed during surgical excision followed by microscopic evaluation immunohistochemistry. Regarding the treatment for SS of the jaw, wide surgical excision is necessary to obtain a clear margin followed by adjuvant radiotherapy, and chemotherapy is the recommended treatment modality (Bergh et al., 1999; Tao et al., 2011; Wushou and Miao, 2015). Primary intraosseous SS of the jaws has a high recurrence rate and tumor-related death (Teixeira et al., 2021). As we can observe from Table 2, three patients died (jaw region). Thus, long-term follow-up is required for early detection of recurrence.

The tongue is the relatively frequently affected intraoral site of SS (Novotny and Fort, 1971; Bridge et al., 1988; Fujimoto et al., 2003; Villaroel-Salinas et al., 2012), and it was first described as a site of SS occurrence by Mir-Abedy (1962). Common signs and symptoms of SS of the tongue include pain in the tongue area, eating difficulties, loss of ability to speak, inability to close lips, hoarseness, frequent choking, sore throat, fever, and swelling. Most cases reported in Table 2 are biphasic subtypes than monophasic, and very few cases are of calcifying SS (Alabdulaaly et al., 2021). In our review, the first case in the tongue area is a monophasic and calcifying subtype (Alabdulaaly et al., 2021). Due to its rarity, diagnosis of the disease is complicated disease. In this specific case, we can observe histologically (monophasic and calcified subtype) that there was an infiltrative tumor composed of a diffuse and highly cellular proliferation of the spindle cells with a ringbone pattern, collagenous stroma, extensive calcification, and numerous dilated vessels (Alabdulaaly et al., 2021). MRI is of significant importance in determining the actual size and location of the tumor in the tongue. As we can observe from Table 2, MRI of the head and neck revealed the presence of a large tumor occupying the left side of the tongue (Su et al., 2018). The tumor was classified as stage IIB based on NCCN guidelines. Tumor of this stage should primarily be managed by *en bloc* resection to obtain oncologically appropriate margins, followed by radiation therapy and adjuvant chemotherapy (Su et al., 2018). After the surgical procedure, the incision healed well and his tongue movement improved. MRI revealed no enlarged lymph nodes in the neck (Su et al., 2018). Treating SS in the tongue includes excision (composite resection of the tonsil and tongue base, radical neck dissection, tracheostomy, and reconstruction *via* transmandibular symphysis osteotomy followed by (with or without) radiotherapy (75 Gy) and chemotherapy (doxorubicin, ifosfamide, and pazopanib) (Engelhardt and Leafstedt, 1983).

### Synovial sarcoma of the paranasal sinus tract

Trible (1970) first described a patient with SS that metastasized to the sphenoid sinus. SS of the sinonasal tract is more common in young adults but can occur at any age, with slight male predominance. The most common signs and clinical symptoms are nasal obstruction, epistaxis, and rhinorrhea. Moreover, the cause of SS arising from the sinonasal tract is unclear (Jain et al., 2018; Lin et al., 2021). Diagnosis of SS of the sinonasal tract remains challenging because of the sheer number of differentials that may present and behave like SS. During diagnosis, the physician should pay attention to some clinical indicators, such as the relatively rapid progression of the symptoms and “disrespecting the anatomical distribution” toward the possible malignant nature of the disease (Subramaniam et al., 2012). Anterior rhinoscopy can be very helpful to determine the exact size and location of the tumor. Ophthalmic examination is also performed in order to determine whether the tumor affects vision and eyeball movements (Jain et al., 2018). CT is also an important tool in verifying whether ethmoid, sphenoid, and frontal sinuses are involved (Jain et al., 2018). In order to avoid misdiagnosis, radiology and clinicopathological features play a significant role in diagnosing SS of the sinonasal tract (Lin et al., 2021). An MRI can be beneficial in the determination of the nature of the defect as it can demonstrate a heterogeneously enhancing lesion along with the surrounding structures, and CT can indicate the nature of the nasal mass (Tateishi et al., 2004).

Moreover, the histopathological study generally confirms the diagnosis, and the primary treatment is complete surgical excision including craniotomy, endoscopic resection, and total maxillectomy (Sun et al., 2003; Gallia et al., 2005; Hannoun et al., 2021). Resection with a negative margin is very difficult to achieve in the paranasal sinus, especially endoscopic sinus resection, and due to the complexity of the anatomical structures, the surgeon should try to achieve a precise border resection if possible. The prognosis of the disease can be affected by some factors, such as the size of the tumor, marginal clarity, mitotic activity, neurovascular invasion, and the ki67 proliferation index (Singer et al., 1996). Table 3 shows that one of the patients underwent partial medial maxillectomy; complete ethmoidectomy, except for cribriform plate/anterior cranial fossa resection; and partial middle turbinectomy. After surgery, 3 weeks later, CT and MRI showed inflammation with no gross residual tumor. Due to the absence of tumor-free resection margin and high-grade sarcoma, the patient underwent ifosfamide-based chemotherapy and external beam radiotherapy of 60–65 Gy (Jain et al., 2018). The central problem when adopting this approach is that it can cause cosmetic and functional defects when trying to achieve precise border resection in regions of complex anatomical anatomy in the parasinus region. Despite all this, it has a high recurrence rate within 2 years (Hoffman et al., 2004; Colville et al., 2005; Nielsen et al., 2015), especially in the skull base and paranasal sinus SS (Owosho et al., 2017), depending on the tumor stage, size, and site (Harb et al., 2007). After surgery, it is recommended to perform regular endoscopic and radiological examinations during the follow-up period. Concerning radiology, CT is more advantageous in detecting tumor recurrence but should be advised after 12 weeks after surgery to avoid false positive results (Wong et al., 2014).

**TABLE 3:** Reported cases of synovial sarcoma in the paranasal sinus tract.

Reference	Age/sex	Location	Size (cm)	Tumor stage	Type	Signs and symptoms	Treatment	Follow-up	Outcome
Sun et al. (2003)	54/M	Maxillary sinus	Unk	Unk	Biphasic	Right-sided nasal obstruction and cheek pressure for 5 weeks	S (total maxillectomy)	NED (45 mo)	
Jain et al. (2018)	36/F	Ethmoid	2 × 2 × 2.5	T2N0M0	Monophasic; negative	Left nasal obstruction and epistaxis since 3 months	Endoscopic excision followed by radiotherapy	Postoperative radiotherapy (IMRT) 45Gy/25 fractions and boost 20Gy/10 fractions to the tumor region; no distant metastasis	NED (6 mo)
Lin et al. (2021)	79/F	Nasal cavity, ethmoid, and frontal and maxillary sinus	Unk	Unk	Monophasic	Nasal obstruction, epistaxis, hyposmia, and exophthalmos	S + C + R	8–40 mo imaging follow-up	Tumor recurred within 2 years
Hannoun et al. (2021)	53/M	Maxillary sinus	3.8 × 2.8 × 4.2	T4aN0M0	Monophasic	Left-sided hearing loss accompanied by a left-sided nasal block and a vague facial and dental pain	S + R	Monthly basis for the first year, and yearly thereafter; with no recurrence 5 mo after therapy	
Wong et al. (2014)	80/F	Ethmoid	Unk	T2N0M0	Monophasic	6-mo history of persistent right-sided epistaxis	S (endoscopic recs)	Local recurrence at 3 mo; endoscopic excision followed by R; distant metastasis: brain and neck node	Died at 9 mo
Saito et al. (2018)	53/M	Maxillary sinus	Unk	Unk	Biphasic	Left nose obstruction over the previous couple of years	S + R	Monthly basis for the first year, and yearly thereafter; with no recurrence 5 months after therapy	
Saito et al. (2018)	53/M	Left maxillary sinus	5 × 5.8	Unk	Biphasic	Left nose obstruction over the previous couple of years	C + R	12 mo	Free of disease after 12 mo of treatment
Kartha and Bumpous (2002)	24/F	Ethmoid	6 × 4×3	T4N2aM0	Monophasic		Recs, R (86 Gy)	Recurrence at 1 mo; C and RT (8,600 cGy) after recurrence; distant metastasis; pulmonary, brain	Died at 9 mo
Dhiman et al. (2021)	43/F	Ethmoid	Unk	Unk	Unk	Progressive nasal obstruction and swelling in the medial canthal region on the left side for the last 4 mo	Tumor-free Resection + ifosfamide-based C + R of 60–65 Gy	Patient remained asymptomatic for 1 year, but later, she was lost to follow-up	6 mo later, a tumor recurrence in the left orbit
Dhiman et al. (2021)	82/F	Ethmoid	Unk	Unk	Unk	Right nostril obstruction, epistaxis, external nasal deformity associated with swelling of the right upper eyelid, double vision, and headache	R based on advanced stage and age of the patient, + ifosfamide-based C	Died after 6 weeks of diagnosis	Died after 6 weeks of diagnosis
Subramaniam et al. (2012)	45/M	Sphenoid	3.3	Unk	Unk	Epistaxis and blurred vision for a few weeks	S (biopsy)	Unk	Unk
Gallia et al. (2005)	44/M	Frontal	3.3 × 2.5	Unk	Monophasic	Right-sided proptosis	S (craniotomy)	2 mo	NED (2 mo)
Ulusan et al. (2005)	52/F	Nasal Septum	1.5 × 1.5	Unk	Unk	Progressively enlarging mass in her left nasal cavity of 1 year duration	S (recs)	18 mo	NED

Unk, unknown; Recs, resection; S, surgery; R, radiotherapy; C, chemotherapy; mo, month; NED, no evidence of disease.

### Synovial sarcoma of the orbital and ocular region

Orbital and ocular SS is exceedingly rare. Table 4 shows common signs and symptoms of patients suffering from SS of the orbital and ocular region: growth of the mass, painless exophthalmia, orbital swelling, proptosis, inflamed conjunctiva, limited ocular motility, and painful cystic lesion. In the ocular region, ophthalmologic examination is of great importance in order to analyze the exact size and location of the lesion (Portelli et al., 2019). However, we should also be clear about the differential diagnosis before making a definite conclusion. (Table 4) In our reported case, the histological differential diagnosis in the last case includes malignant peripheral nerve sheath tumor (MPNST), leiomyosarcoma (LMS), fibrosarcoma (FS), and solitary fibrous tumor (SFT) (Portelli et al., 2019). The cells were in a sheet arrangement and vague fascicles, with alternating hypercellular and hypocellular myxoid areas, and FS-like areas were absent. Similar to synovial sarcoma, malignant peripheral nerve sheath tumors can express S100 protein, EMA, CK7, CK19, TLE1, and SOX10. Furthermore, CD34 is absent in SS. In the last case reported, the neoplasm was negative for CK7, CK19, SOX10, CD34, and muscular markers. Cell fascicles were more prolonged, loose, and ample than intersecting.

**TABLE 4:** Reported cases of synovial sarcoma of the orbital and ocular region.

Reference	Age/sex	Location	Signs and symptoms	Type	Treatment	Follow up
Portelli et al. (2019)	24/F	Left superonasal orbital region	1 year history of gradually growing occasionally painful cystic lesion in the left superonasal orbital region	Monophasic	Surgical intervention with a complete removal of the mass	The patient is free of recurrences and metastases after 20 mo
Votruba et al. (2002)	29/M	Conjunctiva, medial canthus, and right eye. 1-cm mass adherent to the medial rectus muscle	4-mo-hst of growing mass in the conjunctiva	Monophasic	Complete excision with close margins	Unk
Hartstein et al. (2006)	14/M	Left orbit and inferotemporally	1-mo-hst of painless proptosis of the left eye	Biphasic	Total exenteration	Recover and without recurrences after 18 mo
Ratnatunga et al. (1992)	21/F	Left orbit: 3 cm × 1.5 cm × 1.5 cm subconjunctival mass, adherent to the medial rectus muscle and extending posteriorly into the retrobulbar region	5-year-hst of progressively enlarging, painless mass, and limited adduction	Biphasic	Incomplete excision	Unk
Ratnatunga et al. (1992)	42/F	Left orbit: about 2-cm mass, adherent to the tendon sheath of the superior oblique muscle	8-mo-hst of left-sided orbital swelling	Biphasic	Unk	Unk
Shukla et al. (2003)	32/F	Left orbit: 4 × 2.5-cm firm, fixed mass, encroaching up to the cornea	5 years of recurrent swelling and pain around her left eye, diplopia, and VA: 6/60	Biphasic	Partial excision; adjuvant RT.	Unk
Liu et al. (2012)	1.5/F	Left upper eyelid, nasal portion, and adherent to medial rectus muscle	1 mo of painless swelling in her left eyelid, proptosis, inflamed conjunctiva, and limited ocular motility	Poorly differentiated	Orbitotomy; adjuvant CT.	No recurrences and mts after 1 year
Ito et al. (2015)	48/F	Left eyeball, intraocular, behind the iris, 1.5 cm × 1 cm × 0.7 cm	2 mo of left eye visual defect; retinal detachment surgery, vitrectomy, and insertion of silicon explant 24 years before	Poorly differentiated	Enucleation of the eye	Tumor-free after 6 mo
Stagner et al. (2017)	31/F	Left inferior orbit	Fullness in her temporal left lower eyelid, periorbital pain, and hemifacial pain for more than a decade; proptosis, upward displacement of the globe, and eyelid proptosis	Poorly differentiated	Subtotal excision	Recover after 1 year
					Adjuvant RT.	
Xu and Chen, (2017)	6/F	Right orbit and temporal portion	1 week of gradual painless proptosis of right eye	Monophasic	Total excision	Recover after 1 year
					Adjuvant CT.	

Unk, unknown; Recs, resection; S, surgery; R, radiotherapy; C, chemotherapy; mo, month; NED, no evidence of disease.

Referring to Table 4, surgical excision (orbitotomy, partial, and total enucleation of the eye) is the most effective treatment for the disease (Ito et al., 2015). In some cases, like case 2 (Votruba et al., 2002), complete excision was performed, but in other cases, complete resection is not possible due to the complex anatomical structure around the orbital region (Ratnatunga et al., 1992). Sometimes, the patients undergo two surgical interventions due to the complexity of the disease. In one of the following cases, the lesion was partially removed because complete removal was not possible and sent for definitive histopathologic examination. Histological examination showed a spindle cell proliferation, composed of relatively small and fairly uniform metastases. Then, a second surgical intervention with a complete removal of the mass was then undertaken, without ocular complications. The patient is free of recurrences and metastases after 20 months of follow-up (Portelli et al., 2019). In some cases as reported in Table 4, radiotherapy and chemotherapy are also used after surgical excision of SS to minimize the recurrence rate of the disease (Shukla et al., 2003; Hartstein et al., 2006; Liu et al., 2012; Stagner et al., 2017; Xu and Chen, 2017).

### Synovial sarcoma in other rare sites of the head and neck (occipital and salivary gland)

Synovial sarcoma of the suboccipital region is a sporadic condition in patients. As we can observe from Table 5, a 12-year-old girl presented to the hospital with a painless tumefaction on the right suboccipital region evolving for 5 months (Karydakis et al., 2018), and after undergoing imaging examination, MRI showed an encapsulated spindle formation in the region of the soft tissues of the right suboccipital region, and CT showed that there was only thinning of the bone without erosion. Laboratory results did not show specific features, and the alkaline phosphatase level elevated slightly. The little girl underwent tumor resection. She was in the left lateral position in the operating room and underwent a combination of blunt and sharp dissection. The mass was well-circumscribed with a stiff consistency. The bone underneath the lesion was eroded. Furthermore, the lesion was fibrous and viscous. A few days later, after the surgery, the patient was discharged. Complete surgical excision is the primary treatment for SS of the suboccipital region, and as we can notice, it is tough to diagnose as the condition is infrequent.

**TABLE 5:** Report cases of primary synovial sarcoma in other rare sites of the head and neck.

Reference	Age/sex	Location	Signs and symptoms	Size (cm)	Type	Therapy	Follow up
Massarelli et al. (1978)	19/M	Palate	Difficulty in swallowing	1.5	Unk	S + R	Alive 1 year
Jay et al. (2008)	35/F	R parotid	Swelling of the right parotid region of uncertain duration with 10 years history of right-sided facial pain and headache	5 × 3	Monophasic	R total conservative parotidectomy	36 mo alive
Ploutarchos (Karydakis et al., 2018)	12/F	Right suboccipital region	Painless mass for 5 mo	Unk	Unk	Complete S recs	Unk
Mariano et al. (2012)	18/M	R submandibular gland	Mass of the right submandibular region that had been present for the past 12 mo	10 × 8	Monophasic	S + R 70 Gy	10 mo, alive

Unk, unknown; Recs, resection; S, surgery; R, radiotherapy; C, chemotherapy; mo, month; NED, no evidence of disease.

Ultrasonography of the neck is a very important step in the gland region to determine the exact location and size of the lesion. For the diagnosis, the patient underwent fine-needle aspiration biopsy. Table 5shows that a women of age 35 with swelling of the right parotid region of uncertain duration with 10 year history caused by right-sided facial pain and headache was treated with total conservative parotidectomy and was alive for 36 months (Jay et al., 2008). In another case, a young man with mass of the right submandibular gland region that had been present for the past 12 months underwent surgical resection of the submandibular gland and adjuvant radiotherapy to a total dose of 7,000 cGy to prevent recurrence (Mariano et al., 2012). The treatment was effective, with no evidence of the disease. Synovial sarcoma in the palatal region can cause difficulty in swallowing, and the main treatment is usually surgical excision followed by radiotherapy (Sharma and Mehan, 2019).

## Discussion

The reviewed literature demonstrates reported cases of SS in different areas in the head and neck region and patients’ diagnosis, treatment method, and prognosis. SS is generally considered a high-grade sarcoma, marked by a poor prognosis. The tumor site can also affect prognosis, with a worse outcome for tumors arising from anatomic sites other than the extremities. Some cases, like SS in the suboccipital region, are even more uncommon, making it much more complicated in terms of diagnosis and treatment.

From this review, we can say that diagnosing SS in the head and neck region is not easy and can be easily misdiagnosed. SS is divided into subtypes: biphasic, monophasic, and poorly differentiated. Nevertheless, as we can observe from Table 2, there are more monophasic subtypes such as monophasic and calcified subtype. Concerning the diagnosis of SS, microscopically, it is not too difficult to diagnose the biphasic subtype of SS but tough to diagnose the monophasic form. In such cases, immunohistochemistry along with molecular studies can be very effective in the diagnosis. We can also conclude that the poorly differentiated subtype is the most challenging diagnosis, mainly when SS occurs in an unusual location. Cytogenetics can also be very useful in the diagnosis of SS. About 90% of SS harbor a specific translocation between the SYT gene on chromosome 18 and either the SSX1 or SSX2 gene on chromosome X. t (X; 18) (p11.2; q11.2) (Bridge et al., 1988; Bellakhdhar et al., 2018). This translocation results in the fusion of SYT genes located on chromosome 18 with highly homologous SSX-1 (SYT-SSX-1), SSX-2 (SYT-SSX-2), and SSX-4 (SYT-SSX-4) genes on the X chromosome (Pai et al., 1993). Genetic testing effectively distinguishes SS from spindle cells and round-cell sarcomas in poorly differentiated tumors. As mentioned in the review, imaging such as CT and MRI is also essential in diagnosing. Sometimes in some cases where the tumor lesion is tiny, the tumor can be misdiagnosed, but it can also be very helpful in determining the nature of the defect; MRI can show an image of the lesion with the invasion of the surrounding structures and the exact size and location of the lesion. However, microscopic and immunohistochemical examination remains the definitive diagnosis for SS in the head and neck region (Eilber and Dry, 2008).

Concerning the treatment of SS in different areas in the head and neck region, we can conclude that surgical excision is the primary treatment modality (Fiore et al., 2021). Surgical treatment includes complete tumor resection, partial resection, cervical lymph node dissection, and flap reconstruction (Bertolini et al., 2003; Wang et al., 2020). As shown in Table 2, surgical resection with negative margins remains the primary mainstay therapy in patients suffering from SS of the TMJ region. In general, wide surgical resection is the first choice for treating SS of the TMJ. There is no standard protocol for the treatment of SS (Bertolini et al., 2003). Wide local excision is usually recommended, and also adjuvant radiation with or without chemotherapy (de Almeida-Lawall et al., 2009). For patients who underwent partial excision or surgical resection with surgical margin involved by the tumor, adjuvant radiotherapy or chemotherapy can be given (Bilgic et al., 2003; Fiore et al., 2021). Radiotherapy has been reported to be effective in controlling the disease (Artico et al., 2004; Wushou and Miao, 2015; Madabhavi et al., 2018), and while chemotherapy effectiveness is controversial, it can be helpful in treating distant metastasis (Vining et al., 2017; Fiore et al., 2021). Table 1 shows that most patients were treated with surgical resection followed by radiotherapy. The role of radiotherapy in treating laryngeal SS is contentious. The complex anatomy, such as vital vascular structures and nerves within the head and neck region, generally compromises the complete surgical excision, and sometimes resection is associated with high positive margins. Radiotherapy has thus been used as an adjunct to surgery to achieve locoregional disease control (Shein et al., 2021). Harb et al., 2007 found that radiotherapy is associated with lower recurrence rates and higher survival, although the results did not achieve significance. Recent studies demonstrate that patients who did not receive radiotherapy showed a worse prognosis than those who did. Naing et al. (2015) reported that radiotherapy is strongly associated with OS and DSS.

In general, in order to optimize overall treatment outcomes, a multidisciplinary, patient-centered approach must be adopted. Few reported cases concerning the specialist care delivered by allied health services include speech pathology, physiotherapy, and psychology. Surgical excision of the larynx and laryngeal dysfunction is associated with reduced quality of life and overall emotion of the patient, especially in young patients suffering from SS of the larynx. Comprehensive airway rehabilitation and psychological support are essential for these patients (DPC and MacGregor, 2012). Usually, after surgery, follow-up is essential to monitor the recurrence rate and increase disease-free survival (Fonseca et al., 2014). Mamelle et al. (1986) reported that postoperative radiotherapy could help reduce local recurrence and is not associated with any improvements in long-term survival. Table 3 shows that, as reported by Wong et al. (2014), the patient declined postoperative radiotherapy treatment, and after 3 months, the disease recurred. Later, the patients underwent surgical excision of the tumor with postoperative radiotherapy. The patients died after 9 months due to metastasis of the brain and neck nodes. Radiotherapy is beneficial in controlling the disease (Jain et al., 2018).

Chemotherapy has been found to have a particular effect on the treatment of HNSS in one case. Santoro et al. (1995) mentioned disease-free and overall survival improvements with ifosfamide and doxorubicin. Nevertheless, due to lack of data, it is tough to figure out the effect of chemotherapy. Sarcoma Meta-Analysis Collaboration found that adjuvant chemotherapy for adults’ localized resectable soft tissue sarcoma improved time to local recurrence and distant recurrence and overall recurrence-free survival (Adjuvant chemotherapy for localised resectable, 1997; Desar et al., 2018). The effect of chemotherapy on pediatric patients is well-defined and has a high response rate (Venkatramani et al., 2021), whereas in adults, the data are insufficient, and its effect is controversial (Al-Hussaini et al., 2011) and is generally defined as low chemosensitive (Trassard et al., 2001; Jacobs et al., 2018). Cytotoxic chemotherapy is often considered in both the neoadjuvant and adjuvant settings for patients with advanced SS. Combined treatment with doxorubicin and ifosfamide represent front-line therapy for SS. The role of chemotherapy is still under refinement and can be considered in patients at high risk of metastasis or in those with advanced disease. Neoadjuvant chemotherapy might be considered in specific situations, for example, as induction therapy to enhance the outcome of surgery in high-risk sarcoma of extremity. It is sometimes tough to perform surgical interaction in the head and neck region. Surgical resection can also cause some complications related to cosmetic and functional deterioration and increase the risk of perioperative complications. In such cases where surgical option is limited, chemotherapy can be used to decrease the tumor size and make the overall therapy more effective, as shown in the case (Saito et al., 2018) in Table 3. However, the effect of chemotherapy is still very unclear due to lack of data and research (Dhiman et al., 2021). More research should be carried out concerning the effect of chemotherapy on SS. Other research shows that directly targeting the fusion oncoprotein can be used as a new treatment method in SS. As we know, the SS18–SSX fusion gene is consistently retained in synovial sarcoma, and synovial sarcoma cells depend on continued SS18–SSX expression throughout the course of the disease. Therefore, it may be more feasible to target the critical oncogenic pathways to induce the development of SS (Nielsen et al., 2015). Many treatment methods have been identified. However, the impact of these strategies in improving SS outcomes is still limited, thus making current and future research strongly needed to improve the survival of patients with SS.

## Conclusion

This review aims to familiarize ourselves with the diagnosis, treatment modality, and prognosis of SS in the head and neck region. Misdiagnosis of the disease can delay the diagnosis and treatment of SS. Immunohistochemical analysis might be fundamental in diagnosing SS, especially those suffering from the monophasic and poorly differentiated subtypes. The management principle is still unclear; a multidisciplinary approach is essential in managing HNSS. More research must be carried out concerning chemotherapy’s effectiveness in treating SS to improve the survival rate and control the recurrence rate of the tumor.

## References

[B1] Adjuvant chemotherapy for localised resectable soft-tissue sarcoma of adults: meta-analysis of individual data. Sarcoma meta-analysis collaboration. Lancet. 1997;350(9092):1647–1654. 9400508

[B2] Al-Daraji W. Lasota J. Foss R. Miettinen M. (2009). Synovial sarcoma involving the head: Analysis of 36 cases with predilection to the parotid and temporal regions. Am. J. Surg. Pathol. 33 (10), 1494–1503. 10.1097/PAS.0b013e3181aa913f 19623036

[B3] Al-Hussaini H. Hogg D. Blackstein M. E. O'Sullivan B. Catton C. N. Chung P. W. (2011). Clinical features, treatment, and outcome in 102 adult and pediatric patients with localized high-grade synovial sarcoma. Sarcoma 2011, 231789. 10.1155/2011/231789 21559258 PMC3087894

[B4] Alabdulaaly L. AlDawood Z. Afshar S. Rahbar R. Al-Ibraheemi A. Woo S. B. (2021). Calcifying synovial sarcoma of the tongue with SS18 rearrangement: A rare variant in a rare location. Oral Surg. Oral Med. Oral Pathol. Oral Radiol. 132 (5), e186–e189. 10.1016/j.oooo.2020.08.016 32981875

[B5] Allias-Montmayeur F. Durroux R. Dodart L. Combelles R. (1997). Tumours and pseudotumorous lesions of the temporomandibular joint: A diagnostic challenge. J. Laryngology Otology 111 (8), 776–781. 10.1017/s0022215100138617 9327024

[B6] Artico R. Bison E. Brotto M. (2004). Monophasic synovial sarcoma of hypopharynx: Case report and review of the literature. Acta Otorhinolaryngol. Ital. 24 (1), 33–36. 15270432

[B7] Bellakhdhar M. Cheniti A. Ghammem M. Bdioui A. Mestiri S. Meherzi A. (2018). Laryngeal synovial sarcoma: Report of 2 cases. J. Egypt Natl. Canc Inst. 30 (4), 173–176. 10.1016/j.jnci.2018.10.002 30482506

[B8] Bergh P. Meis-Kindblom J. M. Gherlinzoni F. Berlin O. Bacchini P. Bertoni F. (1999). Synovial sarcoma: Identification of low and high risk groups. Cancer 85 (12), 2596–2607. 10.1002/(sici)1097-0142(19990615)85:12<2596:aid-cncr16>3.0.co;2-k 10375108

[B9] Bertolini F. Bianchi B. Pizzigallo A. Tullio A. Sesenna E. (2003). Synovial cell sarcoma of the neck. Case report and review of the literature. Acta Otorhinolaryngol. Ital. 23 (5), 391–395. 15108491

[B10] Bilgic B. Mete O. Ozturk S. A. Demiryont M. Keles N. Basaran M. (2003). Synovial sarcoma: A rare tumor of larynx. Pathol. Oncol. Res. 9 (4), 242–245. 10.1007/BF02893385 14688831

[B11] Bridge J. A. Bridge R. S. Borek D. A. Shaffer B. Norris C. W. (1988). Translocation t(X;18) in orofacial synovial sarcoma. Cancer 62 (5), 935–937. 10.1002/1097-0142(19880901)62:5<935::aid-cncr2820620514>3.0.co;2-e 2842027

[B12] Bukawa H. Kawabata A. Murano A. Ono K. Ogawara K. Shiiba M. (2007). Monophasic epithelial synovial sarcoma arising in the temporomandibular joint. Int. J. Oral Maxillofac. Surg. 36 (8), 762–765. 10.1016/j.ijom.2007.02.014 17433623

[B13] Capelli M. Bertino G. Morbini P. Proh M. Falco C. E. Benazzo M. (2007). CO2 laser in the treatment of laryngeal synovial sarcoma: A clinical case. Tumori 93 (3), 296–299. 10.1177/030089160709300313 17679468

[B14] Carrillo R. Rodriguez-Peralto J. L. Batsakis J. G. (1992). Synovial sarcomas of the head and neck. Ann. Otol. Rhinol. Laryngol. 101 (4), 367–370. 10.1177/000348949210100415 1314035

[B15] Colville R. J. Charlton F. Kelly C. G. Nicoll J. J. McLean N. R. (2005). Multidisciplinary management of head and neck sarcomas. Head. Neck 27 (9), 814–824. 10.1002/hed.20232 16086411

[B16] Crowson M. G. Lalich I. Keeney M. G. Garcia J. J. Price D. L. (2015). Clinicopathologic factors and adjuvant treatment effects on survival in adult head and neck synovial cell sarcoma. Head. Neck 37 (3), 375–380. 10.1002/hed.23605 24430934

[B17] Danninger R. Humer U. Stammberger H. (1994). Synovial sarcoma, a rare tumor of the larynx. Case report and differential diagnostic considerations. Laryngorhinootologie 73 (8), 442–444. 10.1055/s-2007-997169 7945664

[B18] de Almeida-Lawall M. Mosqueda-Taylor A. Bologna-Molina R. E. Dominguez-Malagon H. R. Cano-Valdez A. M. Luna-Ortiz K. (2009). Synovial sarcoma of the tongue: Case report and review of the literature. J. Oral Maxillofac. Surg. 67 (4), 914–920. 10.1016/j.joms.2008.08.031 19304058

[B19] de Araujo V. C. Monteiro D. C. (1989). Oral synovial sarcoma: Report of a case. J. Oral Maxillofac. Surg. 47 (9), 1001–1003. 10.1016/0278-2391(89)90389-3 2547918

[B20] De Logu F. Ugolini F. Caporalini C. Palomba A. Simi S. Portelli F. (2020). TRPA1 expression in synovial sarcoma may support neural origin. Biomolecules 10 (10), 1446. 10.3390/biom10101446 33076385 PMC7602570

[B21] Desar I. M. E. Fleuren E. D. G. van der Graaf W. T. A. (2018). Systemic treatment for adults with synovial sarcoma. Curr. Treat. Options Oncol. 19 (2), 13. 10.1007/s11864-018-0525-1 29516254 PMC5842271

[B22] Dhiman S. Negi S. Moudgil S. Thakur J. S. Azad R. K. (2021). Synovial sarcoma of ethmoidal sinus. Surg. J. (N Y). 7 (3), e195–e198. 10.1055/s-0041-1731634 34395871 PMC8354363

[B23] Doubi A. Doubi M. Alzaher N. Tulbah A. (2019). Synovial sarcoma of the hard palate: The third case in the medical literature. Hematol. Oncol. Stem Cell Ther. 12 (1), 60–63. 10.1016/j.hemonc.2016.12.005 28183682

[B24] Dpc C. MacGregor F. B. (2012). Laryngeal synovial cell sarcoma in an 11 year old boy: Challenges of management and rehabilitation. Int. J. Pediatr. Otorhinolaryngology Extra 7 (3), 97–99. 10.1016/j.pedex.2012.02.002

[B25] Eilber F. C. Dry S. M. (2008). Diagnosis and management of synovial sarcoma. J. Surg. Oncol. 97 (4), 314–320. 10.1002/jso.20974 18286474

[B26] Engelhardt J. Leafstedt S. W. (1983). Synovial sarcoma of tonsil and tongue base. South Med. J. 76 (2), 243–244. 10.1097/00007611-198302000-00025 6297098

[B27] Ferlito A. Gale N. Hvala A. Masera A. HvAlA A. (1981). Synovial sarcoma of the soft palate in a child: A light and electron microscopic study. J. Laryngol. Otol. 95 (2), 197–204. 10.1017/s0022215100090605 6257813

[B28] Fiore M. Sambri A. Spinnato P. Zucchini R. Giannini C. Caldari E. (2021). The Biology of synovial sarcoma: State-of-the-Art and future perspectives. Curr. Treat. Options Oncol. 22 (12), 109. 10.1007/s11864-021-00914-4 34687366 PMC8541977

[B29] Fonseca A. S. Azevedo A. C. Magalhaes F. M. Andrade N. A. (2014). Synovial sarcoma in head and neck: A case report. Int. Arch. Otorhinolaryngol. 18 (1), 87–89. 10.1055/s-0033-1361081 25992071 PMC4296938

[B30] Fujimoto M. Hiraga M. Kiyosawa T. Murakami T. Murata S. Ohtsuki M. (2003). Complete remission of metastatic clear cell sarcoma with DAV chemotherapy. Clin. Exp. Dermatol 28 (1), 22–24. 10.1046/j.1365-2230.2003.01109.x 12558622

[B31] Gallia G. L. Sciubba D. M. Hann C. L. Raman S. P. Westra W. H. Tufaro A. P. (2005). Synovial sarcoma of the frontal sinus. Case report. J. Neurosurg. 103 (6), 1077–1080. 10.3171/jns.2005.103.6.1077 16381195

[B32] Gopalakrishnan V. Amini B. Wagner M. J. Nowell E. N. Lazar A. J. Lin P. P. (2017). Synovial sarcoma of the head and neck: A single institution review. Sarcoma 2017, 2016752. 10.1155/2017/2016752 28655993 PMC5474548

[B33] Granowetter L. Ladas E. Taromina K. Rooney D. Kelly K. M. (2006). Integrative tumor board: Pediatric synovial sarcoma. Integr. Cancer Ther. 5 (1), 48–55. 10.1177/1534735405285950 16578909

[B34] Grayson W. Nayler S. J. Jena G. P. (1998). Synovial sarcoma of the parotid gland. A case report with clinicopathological analysis and review of the literature. S Afr. J. Surg. 36 (1), 32–34. 9601830

[B35] Hannoun B. Hannoun I. Bara A. Alassaf A. Chatty E. M. (2021). Synovial sarcoma of the maxillary sinus - a rare condition managed with a rationalized surgery. Ann. Med. Surg. (Lond). 67, 102538. 10.1016/j.amsu.2021.102538 34276985 PMC8267481

[B36] Harb W. J. Luna M. A. Patel S. R. Ballo M. T. Roberts D. B. Sturgis E. M. (2007). Survival in patients with synovial sarcoma of the head and neck: Association with tumor location, size, and extension. Head. Neck 29 (8), 731–740. 10.1002/hed.20564 17274049

[B37] Hartstein M. E. Silver F. L. Ludwig O. J. O'Connor D. M. (2006). Primary synovial sarcoma. Ophthalmology 113 (11), 2093–2096. 10.1016/j.ophtha.2006.04.037 17074567

[B38] Herrero Laso J. L. Varela Duran J. (1998). Oropharyngeal synovial sarcoma. Report of one case. Otorrinolaringol Ibero Am 25 (4), 353–359. 9707757

[B39] Hirsch R. J. Yousem D. M. Loevner L. A. Montone K. T. Chalian A. A. Hayden R. E. (1997). Synovial sarcomas of the head and neck: MR findings. AJR Am. J. Roentgenol. 169 (4), 1185–1188. 10.2214/ajr.169.4.9308488 9308488

[B40] Hoffman H. T. Robinson R. A. Spiess J. L. Buatti J. (2004). Update in management of head and neck sarcoma. Curr. Opin. Oncol. 16 (4), 333–341. 10.1097/01.cco.0000127880.69877.75 15187888

[B41] Holtz F. Magielski J. E. (1985). Synovial sarcomas of the tongue base. The seventh reported case. Arch. Otolaryngol. 111 (4), 271–272. 10.1001/archotol.1985.00800060095016 2983653

[B42] Hongzhi Quan M. K. Liu Y. Tang Z. Fang L. (2017). Relapse of synovial sarcoma in head and neck after a six-year disease-free period: A case report and literature review. Int. J. Clin. Exp. Med. 10 (12), 16709–16714.

[B43] Ito J. Suzuki S. Yoshida A. Mori T. (2015). Primary intraocular synovial sarcoma in the post retinal detachment operative state. BMJ Case Rep. 2015, bcr2015209919. 10.1136/bcr-2015-209919 PMC4533628 26250366

[B44] Jacobs A. J. Morris C. D. Levin A. S. (2018). Synovial sarcoma is not associated with a higher risk of lymph node metastasis compared with other soft tissue sarcomas. Clin. Orthop. Relat. Res. 476 (3), 589–598. 10.1007/s11999.0000000000000057 29529647 PMC6260045

[B45] Jain A. Saxena A. Meher R. Khurana N. (2018). Synovial sarcoma of the ethmoid sinus. Eur. Ann. Otorhinolaryngol. Head. Neck Dis. 135 (6), 453–455. 10.1016/j.anorl.2017.10.007 30352776

[B46] Jay A. Hutchison I. Piper K. Farthing P. M. Richards P. S. (2008). Synovial sarcoma presenting as a parotid mass: Case report and review of literature. Head Neck 30 (12), 1654–1659. 10.1002/hed.20822 18327782

[B47] Jayasooriya P. R. Madawalagamage L. N. Mendis B. R. Lombardi T. (2016). Diagnostic approach to synovial sarcoma of the head and neck illustrated by two cases arising in the face and oral cavity. Dermatopathol. (Basel) 3 (1), 13–22. 10.1159/000444876 PMC4868929 27195266

[B48] Jernstrom P. (1954). Synovial sarcoma of the pharynx; report of a case. Am. J. Clin. Pathol. 24 (8), 957–961. 10.1093/ajcp/24.8.957 13197324

[B49] Kaoutar C. Ahmedou A. B. Oukessou Y. Abada R. Mohamed R. Mohamed M. (2021). Aggressive papillary carcinoma of the lateral aberrant thyroide: A case report and review of the LITERATTUREggressive papillary carcinoma of the lateral aberrant thyroide: A case report and review of the literatture. Int. J. Surg. Case Rep. 80, 433–436. 10.1016/j.ijscr.2020.09.092 PMC7522380 32998060

[B50] Kartha S. S. Bumpous J. M. (2002). Synovial cell sarcoma: Diagnosis, treatment, and outcomes. Laryngoscope 112 (11), 1979–1982. 10.1097/00005537-200211000-00013 12439166

[B51] Karydakis P. Mitsios A. Giakoumettis D. Antoniades E. Karagianni A. Sfakianos G. (2018). Primitive synovial sarcoma of suboccipital region in child. J. Surg. Case Rep. 2018 (10), rjy286. 10.1093/jscr/rjy286 30386549 PMC6202503

[B52] Kumar S. Shukla S. Avinash S. Fonseca D. Nemade H. Rao L. (2020). Laryngeal synovial sarcoma: A rare clinical entity. Indian J. Surg. Oncol. 11 (1), 125–127. 10.1007/s13193-020-01097-4 33088147 PMC7534761

[B53] Lin N. Liu X. Zhang F. Pan Y. Qi M. Sha Y. (2021). Sinonasal synovial sarcoma: Evaluation of the role of radiological and clinicopathological features in diagnosis. Clin. Radiol. 76 (1), 78 e1–e78.78.e8. 10.1016/j.crad.2020.08.007 32896427

[B54] Liu K. Duan X. Yang L. Yu Y. Liu B. (2012). Primary synovial sarcoma in the orbit. J. AAPOS 16 (6), 582–584. 10.1016/j.jaapos.2012.09.002 23158553

[B55] Liu Z. Jin S. Fu S. Hu Y. He Y. (2015). Management of the primary intraosseous synovial sarcoma of the jaws: Be careful of the surgical margin. J. Oral Maxillofac. Surg. 73 (3), 550–563. 10.1016/j.joms.2014.10.003 25577454

[B56] Luo C. W. Liu C. J. Chang K. M. (2007). Synovial sarcoma of the temporomandibular joint area: Report of a case. Oral Surg. Oral Med. Oral Pathol. Oral Radiol. Endod. 104 (4), e62–e65. 10.1016/j.tripleo.2007.05.009 17703967

[B57] Machen S. K. Easley K. A. Goldblum J. R. (1999). Synovial sarcoma of the extremities: A clinicopathologic study of 34 cases, including semi-quantitative analysis of spindled, epithelial, and poorly differentiated areas. Am. J. Surg. Pathol. 23 (3), 268–275. 10.1097/00000478-199903000-00004 10078916

[B58] Madabhavi I. Bhardawa V. Modi M. Patel A. Sarkar M. (2018). Primary synovial sarcoma (SS) of larynx: An unusual site. Oral Oncol. 79, 80–82. 10.1016/j.oraloncology.2018.02.016 29496353

[B59] Mahesh K. T. Ponnuswamy I. A. David M. P. Shivhare P. Puttaranganayak M. I. Sinha P. (2013). Synovial sarcoma of the buccal mucosa: A rare case report. Case Rep. Dent. 2013, 938291. 10.1155/2013/938291 23762651 PMC3670524

[B60] Mamelle G. Richard J. Luboinski B. Schwaab G. Eschwege F. Micheau C. (1986). Synovial sarcoma of the head and neck: An account of four cases and review of the literature. Eur. J. Surg. Oncol. 12 (4), 347–349. 3023142

[B61] Mariano F. V. Oliveira Gondak R. da Costa M. V. Correa M. B. Lopes M. A. de Almeida O. P. (2012). Primary synovial sarcoma involving the submandibular gland. Oral Surg. Oral Med. Oral Pathol. Oral Radiol. 114 (1), e61–e65. 10.1016/j.oooo.2011.12.004 22727109

[B62] Massarelli G. Tanda F. Salis B. (1978). Synovial sarcoma of the soft palate: Report of a case. Hum. Pathol. 9 (3), 341–345. 10.1016/s0046-8177(78)80091-4 207632

[B63] Maxymiw W. G. Wood R. E. (1990). Synovial sarcoma of the maxillofacial region with osseous involvement. Case report. Int. J. Oral Maxillofac. Surg. 19 (5), 305–307. 10.1016/s0901-5027(05)80426-6 2175761

[B64] Meer S. Coleman H. Altini M. (2003). Oral synovial sarcoma: A report of 2 cases and a review of the literature. Oral Surg. Oral Med. Oral Pathol. Oral Radiol. Endod. 96 (3), 306–315. 10.1016/s1079-2104(03)00209-9 12973286

[B65] Mir-Abedy M. (1962). Considerations on the base of the tongue and its tumors. (Apropos of a case of synoviosarcoma and a case of neurofibroma). Ann. Otolaryngol. 79, 547–561. 14474399

[B66] Mostafapour S. P. Futran N. D. (2000). Tumors and tumorous masses presenting as temporomandibular joint syndrome. Official J. Am. Acad. Otolaryngology-Head Neck Surg. 123 (4), 459–464. 10.1067/mhn.2000.109662 11020186

[B67] Naing K. W. Monjazeb A. M. Li C-S. Lee L-Y. Yang A. Borys D. (2015). Perioperative radiotherapy is associated with improved survival among patients with synovial sarcoma: A seer analysis. J. Surg. Oncol. 111 (2), 158–164. 10.1002/jso.23780 25176165 PMC4305005

[B68] Nakahira M. Sugasawa M. Morita K. (2013). Monophasic synovial sarcoma of the nasopharynx. Auris Nasus Larynx 40 (4), 413–416. 10.1016/j.anl.2012.07.011 22867523

[B69] Nielsen T. O. Poulin N. M. Ladanyi M. (2015). Synovial sarcoma: Recent discoveries as a roadmap to new avenues for therapy. Cancer Discov. 5 (2), 124–134. 10.1158/2159-8290.CD-14-1246 25614489 PMC4320664

[B70] Nomura F. Kishimoto S. (2014). Synovial sarcoma of the temporomandibular joint and infratemporal fossa. Auris Nasus Larynx 41 (6), 572–575. 10.1016/j.anl.2014.07.001 25199745

[B71] Novotny G. M. Fort T. C. (1971). Synovial sarcoma of the tongue. Arch. Otolaryngol. 94 (1), 77–79. 10.1001/archotol.1971.00770070113014 4326553

[B72] Owosho A. A. Estilo C. L. Rosen E. B. Yom S. K. Huryn J. M. Antonescu C. R. (2017). A clinicopathologic study on SS18 fusion positive head and neck synovial sarcomas. Oral Oncol. 66, 46–51. 10.1016/j.oraloncology.2016.12.021 28249647 PMC5640264

[B73] Pai S. Chinoy R. F. Pradhan S. A. D'Cruz A. K. Kane S. V. Yadav J. N. (1993). Head and neck synovial sarcomas. J. Surg. Oncol. 54 (2), 82–86. 10.1002/jso.2930540206 8412164

[B74] Papaspyrou S. Kyriakides G. Tapis M. (2003). Endoscopic CO2 laser surgery for large synovial sarcoma of the larynx. Otolaryngol. Head. Neck Surg. 129 (6), 630–631. 10.1016/s0194-5998(03)01385-8 14663427

[B75] Portelli F. Pieretti G. Santoro N. Gorelli G. De Giorgi V. Massi D. (2019). Primary orbital synovial sarcoma mimicking a periocular cyst. Am. J. Dermatopathol. 41 (9), 655–660. 10.1097/DAD.0000000000001351 30624245

[B76] Pruszczynski M. Manni J. J. Smedts F. (1989). Endolaryngeal synovial sarcoma: Case report with immunohistochemical studies. Head. Neck 11 (1), 76–80. 10.1002/hed.2880110113 2537802

[B77] Rangheard A. S. Vanel D. Viala J. Schwaab G. Casiraghi O. Sigal R. (2001). Synovial sarcomas of the head and neck: CT and MR imaging findings of eight patients. AJNR Am. J. Neuroradiol. 22 (5), 851–857. 11337327 PMC8174948

[B78] Rao G. V. Sravya T. Sivaranjani Y. Bhat V. R. (2014). Primary biphasic synovial sarcoma of gingiva: Report of a rare case. J. Oral Maxillofac. Pathol. 18 (1), 77–80. 10.4103/0973-029X.131916 24959041 PMC4065452

[B79] Ratnatunga N. Goodlad J. R. Sankarakumaran N. Seimon R. Nagendran S. Fletcher C. D. (1992). Primary biphasic synovial sarcoma of the orbit. J. Clin. Pathol. 45 (3), 265–267. 10.1136/jcp.45.3.265 1313455 PMC495497

[B80] Reilly G. Johnston G. (2010). Obstructing synovial sarcoma in the trachea of a 10 year old boy. Paediatr. Anaesth. 20 (3), 287–288. 10.1111/j.1460-9592.2009.03230.x 20470327

[B81] Saito S. Ozawa H. Ikari Y. Nakahara N. Ito F. Sekimizu M. (2018). Synovial sarcoma of the maxillary sinus: An extremely rare case with excellent response to chemotherapy. Onco Targets Ther. 11, 483–488. 10.2147/OTT.S151473 29416348 PMC5789048

[B82] Santoro A. Tursz T. Mouridsen H. Verweij J. Steward W. Somers R. (1995). Doxorubicin versus CYVADIC versus doxorubicin plus ifosfamide in first-line treatment of advanced soft tissue sarcomas: A randomized study of the European organization for research and treatment of cancer soft tissue and bone sarcoma group. J. Clin. Oncol. 13 (7), 1537–1545. 10.1200/JCO.1995.13.7.1537 7602342

[B83] Sharma V. Mehan R. (2019). Monophasic synovial sarcoma of tongue: A rarest of rare case scenario. Indian J. Otolaryngol. Head. Neck Surg. 71 (1), 585–588. 10.1007/s12070-018-1418-0 31742025 PMC6848476

[B84] Shein G. Sandhu G. Potter A. Loo C. Jacobson I. Anazodo A. (2021). Laryngeal synovial sarcoma: A systematic review of the last 40 Years of reported cases. Ear Nose Throat J. 100 (2), NP93–NP104. 10.1177/0145561319850697 31309846

[B85] Shukla P. N. Pathy S. Sen S. Purohit A. Julka P. K. Rath G. K. (2003). Primary orbital calcified synovial sarcoma: A case report. Orbit 22 (4), 299–303. 10.1076/orbi.22.4.299.17246 14685906

[B86] Singer S. Baldini E. H. Demetri G. D. Fletcher J. A. Corson J. M. (1996). Synovial sarcoma: Prognostic significance of tumor size, margin of resection, and mitotic activity for survival. J. Clin. Oncol. 14 (4), 1201–1208. 10.1200/JCO.1996.14.4.1201 8648375

[B87] Stacchiotti S. Van Tine B. A. (2018). Synovial sarcoma: Current concepts and future perspectives. J. Clin. Oncol. 36 (2), 180–187. 10.1200/JCO.2017.75.1941 29220290

[B88] Stadelmann W. K. Cruse C. W. Messina J. (1995). Synovial cell sarcoma of the temporomandibular joint. Ann. Plast. Surg. 35 (6), 664–668. 10.1097/00000637-199512000-00020 8748354

[B89] Stagner A. M. Jakobiec F. A. Fay A. (2017). Primary orbital synovial sarcoma: A clinicopathologic review with a differential diagnosis and discussion of molecular genetics. Surv. Ophthalmol. 62 (2), 227–236. 10.1016/j.survophthal.2016.09.001 27697479

[B90] Sturgis E. M. Potter B. O. (2003). Sarcomas of the head and neck region. Curr. Opin. Oncol. 15 (3), 239–252. 10.1097/00001622-200305000-00011 12778019

[B91] Su Z. Zhang J. Gao P. Shi J. Qi M. Chen L. (2018). Synovial sarcoma of the tongue: Report of a case and review of the literature. Ann. R. Coll. Surg. Engl. 100 (5), e118–e122. 10.1308/rcsann.2018.0045 29607724 PMC5956602

[B92] Subramaniam M. M. Shuen C. S. Petersson F. (2012). Poorly differentiated synovial sarcoma of the sphenoid sinus: Report of the first case and review of synovial sarcomas of the sinonasal tract. Histopathology 61 (6), 1232–1237. 10.1111/j.1365-2559.2012.04340.x 22958201

[B93] Sultan I. Rodriguez-Galindo C. Saab R. Yasir S. Casanova M. Ferrari A. (2009). Comparing children and adults with synovial sarcoma in the surveillance, epidemiology, and end results program, 1983 to 2005: An analysis of 1268 patients. Cancer 115 (15), 3537–3547. 10.1002/cncr.24424 19514087

[B94] Sun J. J. Rasgon B. M. Wild T. W. Hilsinger R. L. Jr (2003). Synovial cell sarcoma of the maxillary sinus: A first reported case. Otolaryngol. Head. Neck Surg. 129 (5), 587–590. 10.1016/s0194-5998(03)01392-5 14595284

[B95] Tao Q. Qiao B. Wang Y. Hu F. (2011). Diagnosis and treatment of primary synovial cell sarcoma that occurred in the left mandible body: A case report and literature review. Oral Surg. Oral Med. Oral Pathology, Oral Radiology, Endod. 111 (2), e12–e20. 10.1016/j.tripleo.2010.08.025 21169037

[B96] Tateishi U. Hasegawa T. Beppu Y. Satake M. Moriyama N. (2004). Synovial sarcoma of the soft tissues: Prognostic significance of imaging features. J. Comput. Assist. Tomogr. 28 (1), 140–148. 10.1097/00004728-200401000-00024 14716248

[B97] Teixeira L. N. da Cruz E. Z. Rosa A. C. G. Rodrigues A. A. Passador-Santos F. de Araujo V. C. (2021). Primary intraosseous synovial sarcoma in the mandible. Case Rep. Oncol. Med. 2021, 9945591. 10.1155/2021/9945591 34877023 PMC8645409

[B98] Tilakaratne W. M. (2006). Synovial sarcoma of the mandible. J. Oral Pathol. Med. 35 (1), 61–63. 10.1111/j.1600-0714.2005.00375.x 16393257

[B99] Trassard M. Le Doussal V. Hacene K. Terrier P. Ranchere D. Guillou L. (2001). Prognostic factors in localized primary synovial sarcoma: A multicenter study of 128 adult patients. J. Clin. Oncol. 19 (2), 525–534. 10.1200/JCO.2001.19.2.525 11208847

[B100] Trible W. M. (1970). Destructive lesions of the sphenoid. South Med. J. 63 (7), 849–852. 10.1097/00007611-197007000-00023 4316747

[B101] Ulusan S. Kizilkilic O. Yildirim T. Hurcan C. Bal N. Nursal T. Z. (2005). Radiological findings of primary retroperitoneal synovial sarcoma. Br. J. Radiol. 78 (926), 166–169. 10.1259/bjr/67990800 15681333

[B102] Venkatramani R. Xue W. Randall R. L. Wolden S. Anderson J. Lopez-Terrada D. (2021). Synovial sarcoma in children, adolescents, and young adults: A report from the children's Oncology group ARST0332 study. J. Clin. Oncol. 39 (35), 3927–3937. 10.1200/JCO.21.01628 34623899 PMC8660012

[B103] Villaroel-Salinas J. Campos-Martinez J. Ortiz-Hidalgo C. (2012). Synovial sarcoma of the tongue confirmed by molecular detection of the SYT-SSX2 fusion gene transcript. Int. J. Surg. Pathol. 20 (4), 386–389. 10.1177/1066896911424897 22007079

[B104] Vining C. C. Sinnamon A. J. Ecker B. L. Kelz R. R. Fraker D. L. Roses R. E. (2017). Adjuvant chemotherapy in resectable synovial sarcoma. J. Surg. Oncol. 116 (4), 550–558. 10.1002/jso.24688 28580620

[B105] Votruba M. Hungerford J. Cornes P. G. S. Mabey D. Luthert P. (2002). Primary monophasic synovial sarcoma of the conjunctiva. Br. J. Ophthalmol. 86 (12), 1453–1454. 10.1136/bjo.86.12.1453 12446398 PMC1771384

[B106] Wang H. Zhang J. He X. Niu Y. (2008). Synovial sarcoma in the oral and maxillofacial region: Report of 4 cases and review of the literature. J. Oral Maxillofac. Surg. 66 (1), 161–167. 10.1016/j.joms.2007.05.007 18083434

[B107] Wang Y. Zhu F. Wang K. (2020). Synovial sarcoma of the floor of the mouth: A rare case report. BMC Oral Health 20 (1), 5. 10.1186/s12903-019-0961-8 31906928 PMC6945757

[B108] White R. D. Makar J. Jr. Steckler R. M. (1992). Synovial sarcoma of the temporomandibular joint. J. Oral Maxillofac. Surg. 50 (11), 1227–1230. 10.1016/0278-2391(92)90160-2 1328569

[B109] Wigand M. C. Hoffmann T. K. Barth T. F. E. Veit J. (2018). Biphasic synovial sarcoma of the epiglottis: Case report and literature review. Auris Nasus Larynx 45 (3), 617–621. 10.1016/j.anl.2017.06.007 28689931

[B110] Wong H. T. Ho C. Y. Nazarina A. R. Prepageran N. (2014). Synovial sarcoma of the ethmoidal sinus. J. Laryngol. Otol. 128 (11), 1022–1023. 10.1017/S0022215114002151 25274107

[B111] Wushou A. Miao X. C. (2015). Tumor size predicts prognosis of head and neck synovial cell sarcoma. Oncol. Lett. 9 (1), 381–386. 10.3892/ol.2014.2634 25435996 PMC4247063

[B112] Xia S. Chen X. Hu Y. Zhang J. (2020). Biphasic synovial sarcoma with extensive calcification in the temporomandibular joint region: A rare case report and literature review. J. Stomatol. Oral Maxillofac. Surg. 121 (5), 592–598. 10.1016/j.jormas.2020.02.005 32109597

[B113] Xu P. Chen J. (2017). Primary synovial sarcoma of the orbit. Ophthalmol. Eye Dis. 9, 1179172117701732. 10.1177/1179172117701732 28469480 PMC5397295

